# High-Sensitivity High-Throughput Detection of Nucleic Acid Targets on Metasurface Fluorescence Biosensors

**DOI:** 10.3390/bios11020033

**Published:** 2021-01-27

**Authors:** Masanobu Iwanaga

**Affiliations:** Research Center for Functional Materials, National Institute for Materials Science (NIMS), 1-1 Namiki, Tsukuba 305-0044, Japan; iwanaga.masanobu@nims.go.jp

**Keywords:** all-dielectric metasurface, sensor, fluorescence detection, nucleic acid, DNA, RNA, SARS-CoV-2

## Abstract

Worldwide infection disease due to SARS-CoV-2 is tremendously affecting our daily lives. High-throughput detection methods for nucleic acids are emergently desired. Here, we show high-sensitivity and high-throughput metasurface fluorescence biosensors that are applicable for nucleic acid targets. The all-dielectric metasurface biosensors comprise silicon-on-insulator nanorod array and have prominent electromagnetic resonances enhancing fluorescence emission. For proof-of-concept experiment on the metasurface biosensors, we have conducted fluorescence detection of single-strand oligoDNAs, which model the partial sequences of SARS-CoV-2 RNA indicated by national infection institutes, and succeeded in the high-throughput detection at low concentrations on the order of 100 amol/mL without any amplification technique. As a direct detection method, the metasurface fluorescence biosensors exhibit high performance.

## 1. Introduction

Nucleic acids such as DNA and RNA are targets in the next-generation medical diagnoses. Currently, employing next-generation sequencers (NGSs), cancers are tested at the early stage or pre-stage in the gene diagnosis. However, the throughput is low and needs substantial improvement. Further, to widely supply medical services based on gene diagnoses, point-of-cure testing (POCT) techniques are highly preferred. Moreover, in this one year, POCT sensing is under huge demand due to the worldwide infection of SARS-CoV-2. Between 2019 and 2020, numerous studies on SARS-CoV-2 have been already reported [1,2,3]. At present, practical tests on SARS-CoV-2 are now limited to high-precision reverse transcription polymerase chain reaction (RT-PCR)/loop-mediated isothermal amplification or to high-throughput antigen detection with relatively low sensitivity. As is widely known, the PCR technique enables us to access DNA/RNA targets at extremely low concentration, owing to the more than $10^{10}$ order amplification. Within one hour of reaction time, 100 copies of RNA genome in 140 μL are indicated as limit of detection (LOD) of RT-PCR by National Institute for Infectious Diseases, Japan [4]. The LOD is equivalent to 714 copies/mL or $1.19\times 10^{-21}$ mol/mL. Some of the electrochemical sensing studies claimed the LODs for SARS-CoV-2 targets near/above RT-PCR precision without any amplification [5,6]; however, there have seemed no actual electrochemical methods to be released for practical sensing of SARS-CoV-2. Thus, there is still room to explore other sensing techniques to detect the nucleic targets in high-sensitivity and high-throughput manners.

Generally, nucleotide sensing does not need antibody (Ab), so that rapid response is possible for any emergent infection once it is analyzed by the NGSs. Thus, there is demand for nucleic acid-based biosensing techniques that are substantially improved in comparison with DNA microarray, which does not have any detection signal enhancement mechanism.

Fluorescence (FL) detection of biomarkers is an established method in biosensing, being recognized as one of the most efficient techniques among various biosensing methods with potential leading to future medical diagnosis [7]. However, almost all the FL-enhancing platforms reported so far exhibited low reproducibility, due to hot spot strategy to obtain the large FL-intensity enhancement. The hot spot is a local nm-scale spot at which resonant electric field intensity is significantly enhanced, whereas the resonant field becomes drastically weak as the positions from the hot spot become distant. Thus, it was a puzzle to consistently realize both FL-intensity enhancement and high reproducibility. The issue was resolved with introducing plasmon-photon hybrid metasurfaces [8], which have large light absorption band, equivalent to large light emittance [9]; thousand-fold FL-intensity enhancement was obtained, almost independent of excitation light spots [10,11], meaning that the enhanced FL response is uniform.

To date, it was demonstrated that a particular type of all-dielectric metasurfaces can highly enhance FL in a highly reproducible manner [12]. As a biosensor to detect protein molecules such as antigens and Abs, the metasurfaces have exhibited high enough performance in practical configurations including human serum [13].

Originally, metasurfaces have diverse potential and have exhibited various applications, stemming from their designs with large degree of freedom [14,15,16]. Most of metasurfaces intended to manipulate light waves for particular purposes such as focusing [17,18,19,20,21,22,23], polarization/phase control [24,25,26,27,28,29], perfect light absorption [30,31,32,33,34,35], and so on. The application of metasurfaces for FL enhancement has been tried in numerous studies; only a very small portion of the studies exceptionally reported prominent success in the very large FL-intensity enhancement more than 1000-fold to reference non-enhancing substrates [10,11,36,37,38,39,40,41]. Among them, the highest reproducibility (greater than 95%) was found in the plasmon-photon hybrid metasurfaces [10,11] and the silicon-on-insulator (SOI)-nanorod metasurfaces [12,13].

In this article, the proof-of-concept experiment for high-sensitivity and high-throughput DNA detection for nucleic acid targets on the SOI-nanorod all-dielectric metasurfaces is addressed. To our knowledge, this is the first report on nucleic acid FL detection on all-dielectric metasurfaces. The target DNAs were chosen from sequence in RNA genome of SARS-CoV-2 and were detected using FL-labeled probes with the complementary sequences to the targets.

## 2. Results

**Figure 1:**
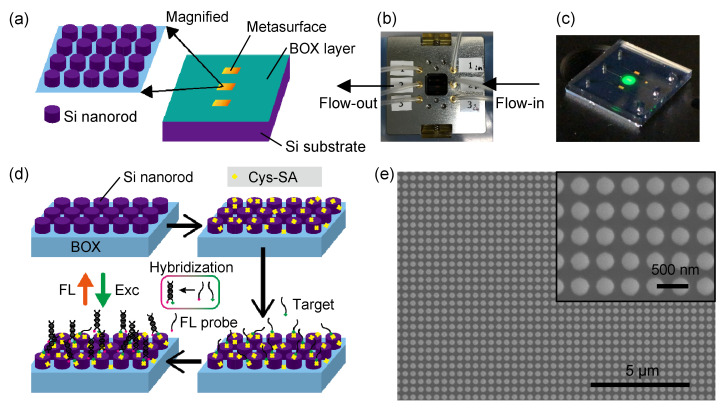
(**a**) Schematic of all-dielectric metasurface structure (left top) and the whole view of metasurface substrate (right). (**b**) Photograph of holder of the metasurface sensor chip. The transparent PDMS chip is 19×19 mm${}^{2}$ in the lateral dimension. (**c**) Metasurface sensor chip in the FL measurement configuration. Green spot is excitation light coming from a LED. (**d**) Schematic of protocol for DNA detection on the metasurface. Cys-SA denotes Cys-streptavidin. (**e**) Top-view SEM images of a SOI-nanorod metasurface. In the wide view, scale bar indicates 5 μm. Inset with scale bar with 500 nm is the magnified view.

Figure 1a provides an appearance of the metasurface substrate. We started SOI substrates, went through nanofabrication process to produce Si-nanorod array, and reached the SOI metasurfaces. The thickness of SOI and buried oxide (BOX) layers was 200 and 375 nm, respectively. The SOI was crystalline silicon with the (100) surface. The two layers were produced on p-type Si wafer of 675 μm thickness; in total, three-layer structure of SOI/BOX/Si wafer was formed.

In the nanofabrication, the nanopatterning was performed on a negative electron beam resist (NEB-22A, Sumitomo Chemical, Tokyo, Japan) employing a high-resolution electron beam drawing instrument (JBX-6300FS, JEOL, Tokyo, Japan), and selective dry etching for the SOI layer was carried out using a deep reactive-ion etching instrument (MUC-21 ASE-SRE, Sumitomo Precision Products, Amagasaki, Japan). As a result, the BOX layer was left as it was, without being affected by the dry etching. The BOX layer of 375 nm on Si wafer looks green, as illustrated in Figure 1a (right). The role of the BOX layer is to separate the thin SOI layer from bulk Si wafer, so that the SOI layer works as a light guide in a highly light-confining way. Thanks to the BOX layer, the SOI nanorods have prominent optical resonances, some of which are classified into Mie resonances [42]. It was found so far that the resonances that strongly localize magnetic-field component inside the SOI nanorods contribute to prominent FL-intensity enhancement more that 1000-fold at the maximum, compared to non-enhancing flat Si wafer [12].

The metasurface substrate was attached with polydimethylsiloxane (PDMS) microfluidic (MF) chip in a self-absorption manner; we call the absorbed pair of metasurface substrate and PDMS chip metasurface sensor chip. Figure 1b shows a photograph of holder loading the metasurface sensor chip. The holder is compatible with in-out tubes (Tygon ND-100-80, Cole-Parmer, IL, USA). After flow of a series of liquid reagents, the metasurface sensor chip was taken out from the holder and measured in a FL-detection setup. Figure 1c shows a photograph that a metasurface sensor chip was illuminated from the top with green light peaked at 530 nm; the light came from a light-emitting diode (LED). Emitted FL from the all-dielectric metasurface is detected in the illumination-collection setup [13]. Further details are described in Section 4.

Figure 1d illustrates a series of protocol for immobilization of biomolecules in the proof-of-concept experiment, which provides a close look of the SOI-nanorod metasurface with omitting the MF chip made of PDMS. Outline of the protocol is as follows. First, binding molecules are immobilized on the SOI-nanorod metasurfaces; in the present case, Cys-streptavidin is used as the binding molecules (yellow) because it can bind on the SOI nanorods via a His-tag; the targets at a certain concentration are assumed to have biotin label (green), flowed in the MF path and immobilized on the binding molecules; finally, FL-labeled probes, which are single-strand DNAs (ssDNAs) and complementary to the targets, hybridize with the target on the SOI-nanorod metasurface and are detected via the enhanced FL signals. More specific details for the DNAs, binding molecules, and MF protocol are described in Section 4.1, Section 4.2 and Section 4.3, respectively. We note that the ssDNA targets can be always biotinylated using a commercial labeling kit; therefore, the assumption above is justified. When the hybridization takes place, the temperature of the metasurface sensor chip has to be controlled as the hybridization becomes optimal below the DNA’s melting temperature ($T_{m}$), at which the DNA dissociates into two ssDNAs.

A top-view scanning electron microscopy (SEM) image of a metasurface is shown in Figure 1e; scale bar indicates 5 μm. The inset is the magnified view; scale bar indicates 500 nm. The diameter of the circular SOI nanorods is approximately 270 nm. The periodic length of the square array was set to 400 nm.

Table 1 lists DNAs based on a partial N-gene RNA sequence, chosen by US Centers for Disease Control and Prevention (CDC) [1] and the complementary sequence. Here, for a proof-of-concept experiment, we set one of the ssDNAs to be FL-labeled probe and the other ssDNA to be target, as shown in Table 1. The target has five thymine (T) extension at both sides to allow some space in hybridization. The melting temperature $T_{m}$ was estimated from guanine-cytosine (GC) percent in the single strands, using the following approximated relation [43],

(1) $$T_{m}=81.5+16.6\log_{10}\left[{\mathrm{Na}}^{+}\right]+0.41\times \mathrm{GC}\left(\%\right)-500/L$$

where *L* denotes length of strand and $\left[{\mathrm{Na}}^{+}\right]$ does Na-ion concentration in molar (M). The values of $T_{m}$ were evaluated assuming $[{\mathrm{Na}}^{+}]=50$ mM. Symbols [HEX] and [BIO] are FL-label HEX and biotin, respectively; the HEX has light absorption peak at 535 nm and FL-emission peak at 555 nm [44], and the [BIO] is additive to the 3${}^{\prime }$-end, being specifically biotin-TEG [45]. Table 1 also lists DNAs modeling a N-gene RNA sequence, determined by National Institute of Health, Thailand (NIHT) [1], and the probe for SARS-CoV-2. The values of $T_{m}$ are evaluated using Equation (1).

Using the sets of probe and target in Table 1, the proof-of-concept experiment was conducted in the detection scheme shown in Figure 1d, where the targets are immobilized on the metasurface and FL-labeled probes bind to the targets, yielding the target-detected FL signals. This scheme is doable using a commercial biotin-labeling kit that adds biotin arm(s) to the ssDNA in test liquid including the targets; the probes can be independently prepared in advance to the target samples.

**Figure 2:**
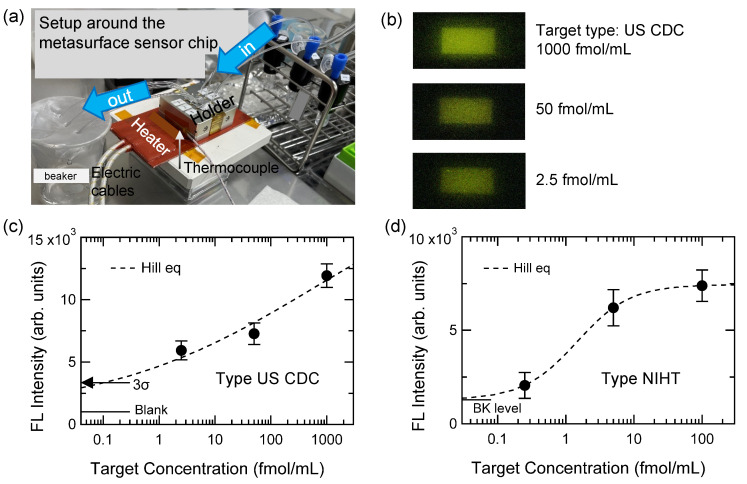
(**a**) Experimental setup around the holder setting metasurface sensor chip. The holder was heated in a feedback manner, as described in the text. (**b**) FL images from the immobilized FL-labeled DNAs (type: US CDC, Table 1). The rectangular metasurface area was 1.2×0.6 mm${}^{2}$ in common. (**c**,**d**) Detection curves of modeled SARS-CoV-2 targets, types of US CDC and NIHT, respectively. Black dashed curves are fitted curves using the Hill equation (Equation (2)). Horizontal bar in subfigure (**c**) indicates target blank (or zero signal) level. An arrow in subfigure (**c**) indicates an estimated LOD level at 3σ (σ: standard deviation) from the blank level. Horizontal bar in subfigure (**d**) indicates background (BK) level, which is equal to $y_{0}$ in Equation (2).

Figure 2a shows a photograph of experimental setup including the holder for metasurface sensor chip, which is seen around the center. When the hybridization is induced in the metasurface sensor chip, the holder was heated. As seen in Figure 2a, the holder was put on a heater covered by silicon rubber (brown); the heater was controlled by a feedback circuit using a thermocouple indicated by a vertical arrow; the thermocouple was fixed at the bottom side of the aluminum holder. The temperature was controlled within 1 °C.

Figure 2b shows a set of FL images, which were measured for the US-CDC-type DNA pairs immobilized on the metasurfaces. The FL images were taken in the configuration shown in Figure 1c. The MF protocol was carried out, as schematically illustrated in Figure 1d. The full descriptions for the MF protocol are provided in Section 4.3. The target concentrations were 2.5, 50, and 1000 fmol/mL from the bottom to the top. The concentration of FL-labeled probes was set to 100 nM in common; this concentration was reasonable because it is not too high and not too low. The FL images are presented with increasing brightness by 40% from the original images. By extracting FL intensities on the metasurface areas of rectangular shapes, Figure 2c shows FL detection curve for US-CDC-type DNA. Dots are the FL intensities and error bars are also indicated, which are mainly ascribed to thermal noise on the charge-coupled device (CCD) camera that does not have any cooling unit. The measured FL data were fitted with the Hill equation, appeared as Equation (2) below; the fitted curve is shown with a dashed curve. From the cross point of the Hill curve and 3σ (σ: standard deviation), which is indicated by a horizontal arrow, from the blank level (horizontal bar), the LOD for the DNA detection is estimated to be 0.11 fmol/mL. We note that the LOD is estimated in a statistical way, based on the experimental σ and blank level.

Let us here remark the relation of standard deviation σ with *p* value. When conducting statistical analysis for biosamples, *p* value is routinely used as biosamples themselves have variance; therefore, it is necessary to evaluate statistical relevance in measuring the samples. However, the samples used for Figure 2b,c were originally purified specimens and quantitatively prepared in a precise manner; therefore, each measured point hardly have variance. We confirmed the high reproducibility in the present MF setup for Ab protein molecules [13]. In the small-variance experimental situations in physics and chemistry, statistical analysis is usually evaluated with the standard deviation σ. We mention that 3σ means p<0.0027.

The Hill equation [46] used in Figure 2c,d has the following form,

(2) $$y=y_{0}+\left(S-y_{0}\right)\frac{x^{n}}{x^{n}+K_{D}^{n}}$$

where *y* is FL-signal intensity; $y_{0}$ is background level without the target; *S* is saturation signal intensity, which was regarded as a proportional constant in fitting; *x* is concentration of target; *n* is degree of cooperative reaction; and $K_{D}$ is dissociation constant [47,48]. Equation (2) is equivalent to the so-called four-parameter logistic equation [13], which is often used to analyze immunoassays. The Hill equation assumes the mass action model for a single chemical reaction under equilibrium [47]; therefore, it can be used not only for protein–ligand binding reaction but also for single-reaction DNA hybridization. The present DNA hybridization in Figure 1d took place at a fixed temperature below $T_{m}$ and followed a single reaction such that

(3) $$D+D_{c}\to H$$

because it did not include any polymerase [49]. In Equation (3), *D* denotes a ssDNA, $D_{c}$ does the complementary ssDNA, and *H* stands for the hybridized DNA. Thus, the present DNA hybridization meets the assumption of the Hill equation. Note that the Hill equation is now understood as an empirical equation that is based on the simple assumption and that fits experimental data in a flexible manner [47]. Trials for comparing the Hill curve with linear and power curves are shown in Supplementary Materials (Figure S1); as a result, it was confirmed that the Hill equation enables to fit the profile quite well.

In Figure 2c, the fitted Hill curve has n=0.23 and $K_{D}=629.2$ fmol/mL. When the degree *n* is less than unity, it suggests that the reaction is anti-cooperative, while the reaction is cooperative for n≥1 [50].

Figure 2d shows FL detection curve for DNAs modeling a partial RNA sequence chosen by NIHT. The target concentrations were 0.25, 5, and 100 fmol/mL (dots); they were detected because they can be experimentally discriminated from the background level indicated by the horizontal bar in Figure 2d. The error bar mainly comes from thermal noise on the CCD camera. Dashed curve shows the Hill curve fitting the measured data; the fitting parameters n=1.1 and $K_{D}=1.44$ fmol/mL. The parameter *n* suggests that the hybridization between the probe and target takes place in a cooperative manner, which plays a positive role in detecting the target at a low concentration of 250 amol/mL of the NIHT-type DNA. It is experimentally possible to discriminate the FL signals at 250 amol/mL from the background level (horizontal bar in Figure 2d). Note that the error bars come not from the sample variance but from thermal noise in the FL image due to the non-cooling CCD camera; therefore, the FL intensity is definitely discriminated from the background level.

**Figure 3:**
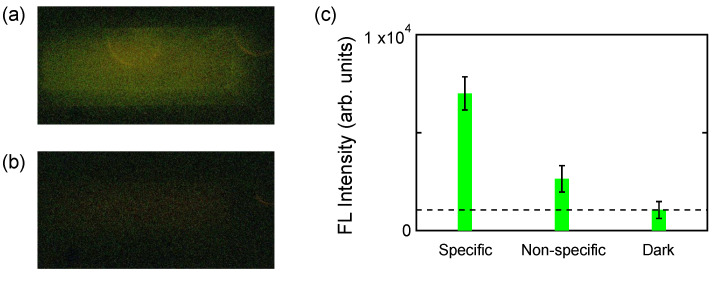
A test of specific detection. FL images of (**a**) specific target–probe pairs and (**b**) non-specific pairs. After the background was subtracted, these images were shown with increasing brightness by 40% and decreasing contrast by −20%. (**c**) Quantitative comparison of FL intensities evaluated from the FL images (**a**,**b**). Dark-level signal is also shown, which originates from dark current on the charge-coupled device (CCD) camera.

Figure 3a,b shows FL images under specific and non-specific conditions, respectively; in Figure 3a, the target and FL-labeled probe were type US CDC in Table 1, whereas, in Figure 3b, the target was type US CDC and the FL-labeled probe was type NIHT in Table 1. We tested specific and non-specific FL signals in the two configurations. The target and FL-labeled-probe concentrations were set to 1 and 50 nM, respectively. The MF-flow protocol is specified in Section 4.3. Obviously, the specific FL image (a) is definitely discriminated from the non-specific one (b). The two images are shown with increasing brightness by 40% and decreasing contrast by −20%.

Figure 3c shows quantitative comparison of the specific and non-specific FL images in Figure 3a,b; the FL intensities were evaluated from Figure 3a,b. We note that two semi-circle patterns appear in Figure 3a, which were avoided to evaluate the FL intensity because the semi-circles are considered to come from bubbles emerged in the PDMS MF chip under the heating condition. Figure 3c shows that ratio of specific data to non-specific data is 2.65; therefore, the signal-to-noise ratio is definite. We consider that the non-specific data is almost background level on the metasurface because the evaluated intensity is almost the same with the intensity flowed with blank liquid. Dark data indicate dark level, which was taken at an area outside the MF paths in the image (b), that is, at an entirely black area; the intensity comes from dark current on the CCD camera.

## 3. Discussion

In this section, we first sum up the experimental results shown in Section 2 and discuss practical situations in which the present metasurface sensors are applicable. Second, we refer to other DNA-sensing results reported so far, mainly in terms of nanostructure platforms, and compare them with the present results.

### 3.1. The Present Metasurface-Sensor Applicable Situation

From the detection concentration ranges in Figure 2, it is evident that the metasurface sensor chips serve as nucleic acid sensors at low concentrations on the order of 100 amol/mL though the order is not at the RT-PCR level. The processing time to detect the targets was about 1 h in total. The setup was compact, including a small CCD camera for FL imaging; thus, the setup meets a POCT-device dimension.

Considering the experimental results and setup, one of the practical situations to use the metasurface sensor chips is rapid nucleic-acid test for SARS-CoV-2. Although the FL-labeled probes need to be replaced with the corresponding oligoRNA sequences, it can be realized in a straightforward way, based on the protocol presented in this article. The nucleic acid test has an advantage to timely respond to mutant SARS-CoV-2, in contrast to the antigen kits using Abs. Considering a large number of the tests, the biotin-trapping pretreatment on the metasurfaces is preferred, similarly to improved commercial immunoassay kits that have microplates precoated with anti-biotin Abs. In addition, optimizations concerning reagents used in the MF protocol will improve the processing time and the signal-to-noise ratio.

### 3.2. Comparison with Other DNA-Sensing Results

Numerous studies have been reported on DNA sensing techniques [51,52]; even when we focus on the studies on ssDNA targets, there is a large number of literature. We here mention two active directions to attain new DNA biosensors. One is based on conjugated polymers [51]; in most of the studies, electrochemical signals were measured for ssDNA detection, and the lower limit of the dynamic ranges takes a very wide range from 1 fM to 50 nM in the twelve cited papers. As we mentioned the electrochemical detection for SARS-CoV-2 in Section 1, very low values are sometimes claimed to be detected with electrochemical techniques. However, to our knowledge, practical electrochemical biosensors with extremely low LOD have not been established. The other intends to exploit aptamers [52]; the studies on aptamer biosensors are rapidly enlarging the targets in these five years. DNA aptamers are designed to have a particular function and can amplified electrochemical or FL signals; therefore, they have potential to contribute to highly sensitive sensing for ssDNA though we have not found any literature exhibiting such results.

Next, we address DNA sensing using artificial nanostructure platforms, instead of ordinary substrates or plastic plates. DNA detections on plasmonic platforms using gold nanostructures have been conducted by many researchers. We first address some of the representative results. FL detection on gold nanodisks was performed using salmon sperm DNA, which is now available in commercial test kits; FL-labeled ssDNA concentrations varied from 5 fM to 5 nM and, after 4 h incubation, FL signals from hybridized pairs of the FL-labeled ssDNA and probe ssDNA were measured [53]; the probe was fixed in advance on a gold nanodisk-dispersed substrate. Although the long-time incubation for the hybridization is necessary, the detected FL signals are quite evident, compared to the reference at 0 M target concentration.

Regarding plasmon-assisted sensing, resonant wavelength shift is a popular technique, which stems from surface plasmon resonance on flat gold surface [54]. Extremely high sensitivity of 100 aM for microRNA (miRNA) was claimed based on the measured resonant wavelength shift on local plasmon resonance [55]; the miRNA detection went through a long process such that cell culture took 6 days, transduction and extraction of the miRNA took further 4 days, the isolation needed more than 100 min, and incubation on gold nanostructure occurred overnight; in total, a week was needed for one run. In contrast, a high-throughput sensing [56] conducted at 1 h for miRNA based on the resonant wavelength shift, surface-bound hybridization chain reaction was incorporated; the LOD was 1 pM. Thus, high throughput and high sensitivity are trade-off. The present all-dielectric metasurface platform needs about 1 h operation time and detects 100 amol/mL-order (that is, 100 fM-order) concentrations; thus, it offers a good method minimizing the trade-off regarding operation time and sensitivity.

In contrast to the plasmonic platforms, all-dielectric platforms have been rarely applied to FL sensing for nucleic acids. As a dielectric nanostructure platform for the FL biosensing, Si nanowires were recently reported [57]; the Si nanowires were densely grown in a chemical way, spending more than 16 h; most of the nanowires contacted with the nearest next-neighbors, the typical height was 3 μm, and the width looks to vary in a wide range from 10 to 500 nm. Thus, the Si nanowires are different from the metasurfaces that are precisely fabricated following artificial designs. It was shown that the Si nanowires work as an ultrasensitive optical biosensor for Hepatitis B virus genome without any amplification or in a PCR-free manner. It was reported that, after 2 h hybridization time of the target genome on the Si nanowires, an extremely low concentration of 20 copies/100 μL was detected.

Antigen kits are currently used for SARS-CoV-2 (e.g., Espline, Fuji Rebio, Tokyo, Japan); the LOD is claimed to be 25 pg/mL. In spite of the low LOD, pseudo-positive results are frequently reported. They seem to be suitable as tests for those who are suspicious of the infection, whereas they are unlikely to be a good option to confirm that persons are negative.

### 3.3. Concluding Remarks

We conducted the proof-of-concept experiment for nucleic acid detection on the metasurface FL biosensors and obtained the FL signals from the target DNA modeling SARS-CoV-2 RNA (type: NIHT) at a low concentration of 250 amol/mL less than 30 min hybridization time. Another the SARS-CoV-2 RNA-modeling DNA (type: US CDC) was also detected and the LOD was estimated to be at 110 amol/mL. Thus, the values of LOD are in an order of 100 fM, being better than the high-throughput optical nucleic-acid-detection methods reported so far.

Although the detection efficiency does not reach that by PCR techniques at present, the metasurface nucleic-acid sensors are a possible alternative solution for rapid screening of the antigen kits of SARS-CoV-2 with modifying the FL probes into the RNA sequence to match the RNA targets. The metasurface sensors will moreover enable us to detect any unknown infection virus with minimum delay, for which we are not ready to produce the corresponding antibody.

## 4. Materials and Methods

### 4.1. OligoDNAs

The FL-labeled DNAs were purified using reversed-phase cartridge column just after the synthesis. The DNAs with biotin end were purified with high-performance liquid chromatography. They were ordered in custom to a company (Eurofin Genetics, Tokyo, Japan) and delivered in lyophilized forms. The oligoDNAs were reconstituted at 100 μM with RNase-free TE buffer, pH 8.0 (TE8.0) (AM9849, ThermoFisher Scientific, Waltham, MA, USA) and aliquoted; the specimens, which were not readily used, were stored at −20 °C.

### 4.2. Binding Protein Molecules

Cys-streptavidin (PRO1005, Click Biosystems, TX, USA) was originally designed to immobilize directly on gold particles. The His-tags form covalent Au–S bonds, enabling the immobilization. The His-tags can also bind to the outmost Si surface made of thin SiO${}_{2}$ [50], so that the Cys-streptavidin is able to immobilize on the SOI-nanorod surface.

### 4.3. MF-Flow Protocol

At the beginning, MF-flow channels in the metasurface sensor chip were filled with phosphate-buffered saline (PBS), pH 7.4 (164-25511, FUJIFILM Wako Pure Chemical, Osaka, Japan). The flow rate of the PBS was always set to be approximately 10 μL/min. The Cys-streptavidin described in the previous Section 4.2 was diluted at 20 μg/mL with the PBS and flowed at 8–10 μL/min for 20 min. After rinsing the MF paths with the PBS for 10 min, the target ssDNAs with biotin ends were flowed at 5±1 μL/min for 30 min. They were diluted with the PBS at intended concentrations. The MF paths were rinsed again with the PBS for 10 min. At this stage, the targets were immobilized on the SOI-nanorod metasurface. To replace the PBS with a mixed buffer of SSC, pH 7.0 (15557044, ThermoFisher Scientific), which was made of 3.0 M sodium chloride and 0.3 M sodium citrate, and of the TE8.0 in the MF paths, the mixed buffer SSC/TE8.0 was flowed for 10 min; the volume ratio of SSC:TE8.0 was 1:2; at the same time, the holder was heated in the configuration shown in Figure 2a. For the type US CDC, the temperature was set to 55 °C and, for the type NIHT, the temperature was set to 45 °C. The temperatures were set based on a criterion of ($T_{m}-5$) °C. The FL-labeled probes were flowed at 100 nM for the type US CDC and 200 nM for the type NIHT. We did not observe definite difference at more than 100 nM. The FL probes were diluted with the mixed SSC/TE8.0 buffer. The flow rates were 15–17 μL/min for the US CDC and 5 μL/min for the NIHT; the time was 15 and 20 min, respectively. When we stopped the flows of the FL probes, the heating finished. Finally, the MF paths were rinsed with the mixed buffer for 10 min, and then the metasurface sensor chip went through FL measurement in the configuration shown in Figure 1c.

In the measurement for Figure 3, the target was flowed at 1 nM concentration for 30 min at flow rate of 5 μL/min in both channels. After rinsing the channels with the phosphate-buffered saline (PBS) for 10 min, the PBS in the MF paths were replaced with the mixed SSC/TE8.0 buffer at 55 °C; the PBS and mixed buffer are specified above. The FL-labeled probes diluted with the mixed buffer to 50 nM were flowed at 55 °C for 10 min in each channel. After rinsing the channels with the mixed buffer for 10 min without heating, we took the FL images.

### 4.4. FL Measurement

FL measurement was conducted under illumination of green LED light, as shown in Figure 1c. A bandpass filter with optical density (OD) 4 and a short pass filter restricted the transmission band of the LED light to a range of 515 to 540 nm. An achromatic lens focused the filtered light on the metasurface sensor chip. The illumination light power was 0.40 mW on the sensor chip surface. The FL emitted from the sensor chip was collected with an achromatic lens, went through two OD4 filters passing only a range of 561 to 621 nm, and reached the CCD camera, on which the exposure time was set to 10 s. The raw FL images were stored in 16-bit TIFF format.

## 5. Patents

The metasurface biosensors employed in this article were filed in a Japan Patent (P2019-231043) and a PCT Patent (PCT/JP2020/041153).

## Figures and Tables

**Table 1:** FL-labeled ssDNA modeling a partial N-gene RNA of SARS-CoV-2 and the complementary probe (type: US CDC) [1]; the GC (%) is 41.2 and 58.3%, respectively. Another set is also shown (type: NIHT) [1]; the the GC (%) of the target and probe is 30.8 and 50.3%, respectively. Symbols [HEX] and [BIO] denote FL-label HEX and biotin, respectively. $T_{m}$ is melting temperature, evaluated using Equation (1).

Type	Role in Sensing	ssDNA Sequences	$T_{m}$ (°C)
US CDC	FL-labeled probe	5${}^{\prime }$-[HEX]GGTCCACCAAACGTAATGCGGGGT-3${}^{\prime }$	63
US CDC	Target	5${}^{\prime }$-TTTTTACCCCGCATTACGTTTGGTGGACCTTTTT[BIO]-3${}^{\prime }$	62
NIHT	FL-labeled probe	5${}^{\prime }$-[HEX]TGGTTACTGCCAGTTG-3${}^{\prime }$	49
NIHT	Target	5${}^{\prime }$-TTTTTCAACTGGCAGTAACCATTTTT[BIO]-3${}^{\prime }$	53

## Data Availability

The data presented in this study are available on request from the corresponding author.

## Supplementary Materials

The following is available online at https://www.mdpi.com/2079-6374/11/2/33/s1. Figure S1: Comparison of the Hill curve with other curves.

## Abbreviations

The following abbreviations are used in this article:

Ab	antibody
aM	attomolar (molar M = mol/L)
BOX	buried oxide
CCD	charge-coupled device
CDC	Centers for Disease Control and Prevention
DNA	deoxyribonucleic acid
FL	fluorescence
fM	femotomolar
GC	guanine-cytosine
LED	light-emitting diode
LOD	limit of detection
MF	microfluidic
miRNA	microRNA
NGS	next-generation sequencer
NIHT	National Institute of Health, Thailand
nM	nanomolar
OD	optical density
PBS	phosphate-buffered saline
PDMS	polydimethylsiloxane
pM	picomolar
POCT	point-of-cure testing
RNA	ribonucleic acid
SEM	scanning electron microscopy
SOI	silicon on insulator
SPR	surface plasmon resonance
ssDNA	single-strand DNA
T	thymine
TE8.0	TE buffer, pH 8.0
