# Supplementary material for: High-Sensitivity High-Throughput Detection of Nucleic Acid Targets on Metasurface Fluorescence Biosensors
Source: Biosensors (Basel). 2021 Jan 27;11(2):33. doi: 10.3390/bios11020033 (PMC7911868; doi:10.3390/bios11020033)
Supplement: Supplementary file 1 [file biosensors-11-00033-s001.pdf]

Supplementary Materials: High-Sensitivity High-Throughput Detection of Nucleic-Acid Targets on Metasurface Fluorescence Biosensors

Masanobu Iwanaga

Research Center for Functional Materials, National Institute for Materials Science (NIMS), 1-1 Namiki, Tsukuba 305-0044, Japan

1. COMPARISON OF FITTING CURVES

To explore possibility for other fitting curves for the experimental data in Figure 2c, we compare two other fitting curves in Figure S1: (a) Linear and (b) power functions. Figure S1a shows the Hill curve (dashed curve), which is the same with the curve in Figure 2c, and the fitted linear function (red line) for the four experimental data points; note that they are shown in the linear scale. Obviously, the Hill curve gives us a better fitting result than the linear function.

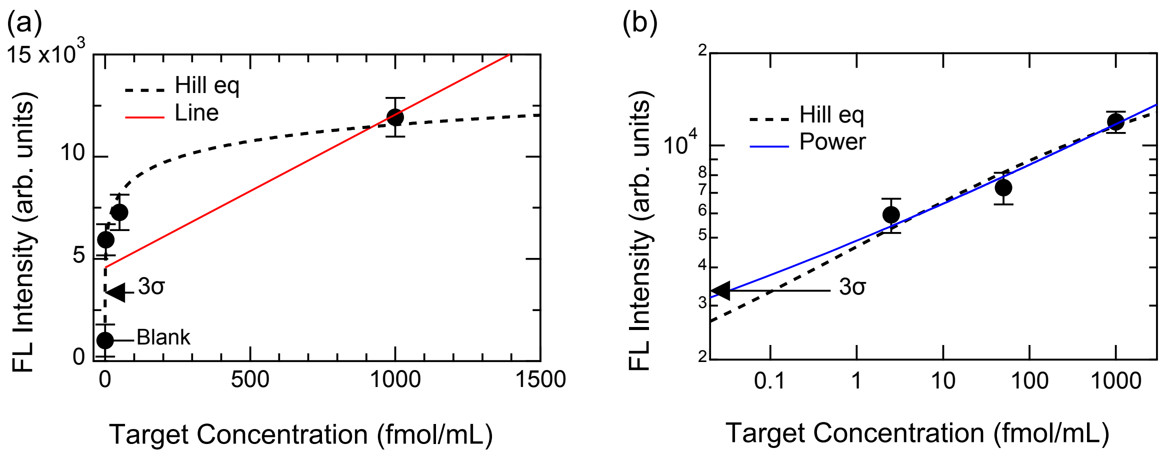

Figure S1. Comparison of the Hill curve (dashed curve) with other curves. Dots with error bars are experimental data, which are the same to those in Figure 2c. The Hill curve is also the same to that in Figure 2c. (**a**) A fitted linear function is shown with a red line. Note that the data and curves are shown in the linear scale. (**b**) A fitted power-law function is shown with a blue line together with the Hill curve in the log-log scale.

Figure S1b shows the Hill curve (dashed curve) again and the fitted power function (blue), which has the form such that $y = A\,x^b + c$ where A is proportional constant and b is power factor. The fitting resulted in $b = 0.147$ ($A = 3885.6$, $c = 1004.2$). Limit of detection (LOD) is estimated to be 0.033 fmol/mL from the cross point of the power-function curve and the 3σ level indicated by a horizontal arrow. The LOD is 3.3-times smaller than the LOD estimated from the Hill

curve (0.11 fmol/mL). Since the model deriving the power function does not seem well established for the single chemical reactions, we regard the LOD by the power function as a reference value. Since the derivation of the Hill equation is well known, we believe that the Hill curve is relatively reliable than the power and linear functions in the fitting analysis. In other words, the DNA hybridization was induced at an equilibrium condition and went through a single chemical reaction; therefore, the Hill equation serves as a fitting function in this study. If DNA hybridizations take place under non-equilibrium (for example, temperature-varying conditions, polymerase-included cases, and so on), there needs, in principle, a different analysis from the Hill equation.
